# Cyclophilins as Modulators of Viral Replication

**DOI:** 10.3390/v5071684

**Published:** 2013-07-11

**Authors:** Stephen D. Frausto, Emily Lee, Hengli Tang

**Affiliations:** Department of Biological Science, The Florida State University, Tallahassee, FL 32306, USA; E-Mails: sfrausto@bio.fsu.edu (S.D.F); elee@bio.fsu.edu (E.L.)

**Keywords:** cyclosporin, HIV, HCV, cyclophilin

## Abstract

Cyclophilins are peptidyl‐prolyl *cis/trans* isomerases important in the proper folding of certain proteins. Mounting evidence supports varied roles of cyclophilins, either positive or negative, in the life cycles of diverse viruses, but the nature and mechanisms of these roles are yet to be defined. The potential for cyclophilins to serve as a drug target for antiviral therapy is evidenced by the success of non-immunosuppressive cyclophilin inhibitors (CPIs), including Alisporivir, in clinical trials targeting hepatitis C virus infection. In addition, as cyclophilins are implicated in the predisposition to, or severity of, various diseases, the ability to specifically and effectively modulate their function will prove increasingly useful for disease intervention. In this review, we will summarize the evidence of cyclophilins as key mediators of viral infection and prospective drug targets.

## 1. Introduction

Unlike other infective agents, viruses do not encode a full complement of proteins that allow them to proliferate independent of the host. Consequently, the life cycles of all viruses depend upon numerous host proteins which may be generally termed host cofactors. Increased understanding of how viruses co-opt host cofactors to generate and maintain a permissive environment for their replication has highlighted targets for antiviral intervention. A substantial body of evidence supports a role in diverse viral life cycles for the cyclophilins, a family of highly conserved peptidyl-prolyl *cis/trans* isomerases.

## 2. Peptidyl-Prolyl *cis/trans* Isomerases (PPIases) and Cyclophilins

Peptide bonds joining adjacent amino acids have partial double bond character, which restricts the free rotations of these bonds. For most peptide bonds, two energetically-preferred states exist, of which the *trans* state is favored due to steric hindrance in the *cis* state. Crossing between these states is energetically costly. In Xaa-Pro peptide bonds, however, the energy minima are more similar between the *cis* and *trans* isomers, resulting in a higher percentage of isomers containing *cis* peptidyl-prolyl bonds. The stereostate of the peptidyl-prolyl bond is critical in many biological processes including signaling, enzyme function, and membrane trafficking. Spontaneous interconversion, however, occurs at a physiologically-impractical rate (*i.e.*, order of seconds), potentially ‘trapping’ proline-containing regions of a protein in a particular conformation. In 1984, a novel enzyme, peptidyl-prolyl *cis/trans* isomerase (PPIase), was isolated that catalyzed this *cis/trans* interconversion, through the use of a target peptide sequence Ala-Ala-Pro-Phe and a chymotrypsin-coupled protease assay. In this peptide, a majority of the peptidyl-prolyl bonds are in the *trans* state, making it a high affinity substrate for the protease. The minority *cis* isomers are poor substrates and are cleaved at a rate determined by the spontaneous isomerization of proline, a rate which is greatly enhanced by the PPIase [1]. In addition, the enzyme was shown to be involved in the refolding of urea-denatured ribonuclease A, a cellular protein [2]. Independent concurrent efforts to identify the cellular receptor for the immunosuppressant cyclosporine A (CsA) led to the discovery of a cytosolic protein with high affinity for CsA [3]. This protein, termed cyclophilin (CyP), would turn out to be the same protein as PPIase [4,5]. In humans, this protein is the 18 kDa cyclophilin A (hCyPA), encoded by the gene PPIA. Human CyPA mediates the immunosuppressive function of CsA through the formation of a CsA/CyPA complex. This complex binds to and inhibits the function of the protein phosphatase calcineurin [6], which normally functions to dephosphorylate NF-AT, a transcription factor important for T cell activation.

Cyclophilin genes are found in the genomes of all domains of life including that of a mimivirus [7,8]. The cyclophilin family is large and has been implicated in various diseases including cancer, diabetes, neurodegeneration, and atherosclerosis (Table 1). They are defined by the presence of a conserved cyclophilin-like domain (CLD) but many also contain additional domains that may function independent of the CLD. Furthermore, some CLDs did not exhibit PPIase activity when tested *in vitro* [9], suggesting that even the single domain cyclophilins may have PPIase-independent roles such as protein chaperoning and quaternary structure regulation. 

Other than cyclophilins, the FK506-binding proteins (FKBPs) also exhibit PPIase activities [10,11]. FK506 is another immunosuppressant that inhibits lymphocyte activation and has been used clinically to facilitate transplant and graft acceptance. Together, cyclophilins and FKBPs comprise the immunophilin family, so named for their binding of the immunosuppressive agents CsA, FK506, and rapamycin. At least thirteen human genes encode proteins containing from one to four FKBP domains. Like cyclophilins, FKBPs exhibit diverse biological functions. The CsA/CyP and the FK506/FKBP complexes exert their immunosuppressive effects through identical mechanisms. Despite the distinct structures between the compounds and between the proteins, the two complexes nevertheless possess a similar composite surface that binds to calcineurin and inhibits T cell activation [6]. The rapamycin/FKBP complex, on the other hand, targets the mammalian target of rapamycin (mTOR) pathway to inhibit T cell proliferation [12,13]. Finally, a non-immunophilin class of PPIase, the parvulin-like proteins, has also been described and includes two human genes encoding at least three distinct proteins [14].

**Table 1:** Diversity of the cyclophilins.

Cyclophilin (*Gene*)	Localization	Size	CsA Binding	Example Cellular Roles
CyPA (*PPIA*) NM_021130	Cytoplasm; Nucleus; Secreted	18 kDa	Y	Inflammation; tumor progression [15,16,17]
CyPB (*PPIB*) NM_000942	ER; Secreted; Cell surface	20 kDa	Y	Secretory pathway; inflammation [18,19,20,21,22]
CyPC (*PPIC*) NM_000943	Cytoplasm; ER; Secreted	33 kDa	Y	Circulating tumor cell survival [23,24]
CyPD [CyP3] (*PPIF*) NM_005729	Mitochondrion	22 kDa	Y	Mitochondrial permeability transition (mTP) [25,26]
CyPE [CyP33](PPIE) NM_006112	Nucleus	33 kDa	Y	mRNA processing [27,28,29,30]
Cyp40 (*PPID*) NM_005038	Cytoplasm	41 kDa	Y	Hsp90 chaperone complex [31,32]
CyPNK(*NKTR)* NM_005385	Membrane	150 kDa	Y	Tumor recognition in NK cells [33,34]
CyPG [SR-cyclophilin] (*PPIG*) NM_004792	Nucleus	88 kDa	Y	Splicing; interaction with RNA pol II [35,36]
CypH [USA-CyP;SnuCyP-20] (*PPIH*) NM_006347	Nucleus; Cytoplasm	19 kDa	Y	mRNA processing; splicing [37,38,39]
CyPL1 (*PPIL1*) NM_016059	Nucleus	18 kDa	Y	mRNA processing [40,41,42]
CyP60 (*PPIL2*) NM_014337	Nucleus; Golgi	59 kDa	N	Cell surface expression of CD147 [43,44]
CyPJ (*PPIL3*) NM_032472	Nucleus	18 kDa	N.D.	mRNA processing [45,46]
PPIL4 (*PPIL4*) NM_139126	Nucleus	57 kDa	N.D.	[47]
PPIL6 (*PPIL6*) NM_173672		35 kDa	N	[9]
RanBP2 [Nup358] (*RANBP2*) NM_006267	Nucleus	358 kDa	N	Nuclear pore complex [48,49]
PPWD1 (*PPWD1*) NM_015342	Nucleus	73 kDa	Y	mRNA processing [27,50]
SDCCAG-10 (*CWC27*) NM_005869	Nucleus	54 kDa	N	[51]

## 3. Cyclophilins and HIV

The first link between viral replication and a host cyclophilin was reported two decades ago by Luban and colleagues, who identified CyPA and CyPB as interaction partners of the HIV-1 Gag polyprotein using a yeast two-hybrid screen [52]. Subsequently it was reported that CyPA, but not CyPB, is specifically incorporated into HIV-1 virions [53,54] via a specific interaction between residues G89 and P90 of the HIV-1 capsid (CA) and the active site of CyPA [55,56]. Mutations in the CyPA-binding site of CA [57] or knockout of CyPA in a T-cell line [58] significantly impaired HIV-1 replication. Although it was initially hypothesized that virion-associated CyPA was required during the HIV-1 life cycle, later studies revealed a role specifically for CyPA present in the target, rather than the producer cells [59,60,61]. HIV virions produced in the presence of CsA or from CyPA-knockdown cells did not exhibit any defect in infecting permissive cells, and wildtype virus produced in the presence of CyPA could not productively infect CyPA-depleted target cells. The mechanism by which CyPA functions to facilitate HIV infection is not fully understood but may be related to the regulation of stability of the incoming viral capsid [61,62,63]. 

In addition to being a proviral factor in HIV-permissive human cells, the ability of CyPA to bind HIV capsid also contributes to an intrinsic immunity mechanism that underlies species-specific restriction of HIV infection. Early attempts to establish HIV infection in non-human primate cell lines revealed a dominant, capsid-dependent block post-entry and immediately prior to reverse transcription in Old World monkeys [64,65,66,67,68,69]. The protein responsible for this restriction has been identified in rhesus macaques to be TRIM5α [70], which binds the incoming viral capsids and halts infection. The human TRIM5α protein only has weak inhibitory action against HIV-1, likely due to sequence divergences in the C-terminal domain of TRIM5α that mediate its recognition of the capsid [70]. An involvement of CyPA in this restriction phenotype was first implicated by the result that CsA treatment of owl monkey cells paradoxically increased HIV infection, presumably counteracting a restriction factor [59]. The discovery of the TRIMCyp fusion gene from owl monkeys [71,72] offered an immediate explanation for this observation: the retrotransposed CyPA domain would target TRIM5α directly to incoming HIV-1 capsids to exert its antiviral activity; and, disruption of CyPA-CA interaction by CsA relieves the restriction. In a remarkable example of convergent evolution, perhaps in defense against pathogens, independent retrotranspositions of CyPA into TRIM5α occurred in several different primate species, resulting in at least three distinct TRIMCyp fusions for which the chimeric protein or transcript is expressed [73,74,75,76]. In addition, for some TRIM5α variants, the restriction function of the unfused TRIM5α is also dependent on CyPA [77,78,79]. In this case, CyPA may modulate CA structure to present a better binding surface for TRIM5α recognition. Regardless of the mechanism, the opposing roles of CyPA in permissive and non-permissive cells, the convergent evolution of TRIMCyp fusion genes, and the multiple ways of using CyPA to gain optimal access to viral capsid all strongly argue for a highly conserved function of CyPA-mediated recognition of viral capsids during the co-evolution of retroviruses and the host cell. Finally, a role of CyPA in HIV pathogenesis is also supported by genome-wide association studies that showed single nucleotide polymorphisms (SNPs) in the regulatory region of the CyPA gene which influence HIV susceptibility [80] or disease progression [81,82], likely through the modulation of CyPA expression. 

Two other HIV proteins, Vpr and p6, have also been reported to interact with CyPA [83,84]. The CyPA-binding site on Vpr was first mapped to a proline-containing peptide at the N-terminus [83], but when full-length Vpr was assayed for interaction, a proline-free region nearer the C-terminus was identified as the site with higher affinity for CyPA, the first such proposed natural binding sequence for CyPA [85,86]. The relevance of CyPA-binding for Vpr function in HIV life cycle (*i.e.*, inducing cell cycle arrest) is controversial [83,87], and it remains to be determined if the p6-CyPA interaction observed *in vitro* also occurs in the infected cells [84]. 

Another cyclophilin that has been implicated in the HIV-1 lifecycle is RanBP2. Also named Nup358, RanBP2 is a large protein containing a C-terminal CLD. It is a component of the nuclear pore complex (NPC), of which HIV relies on to transport replication intermediates. RanBP2 was identified in two genomic siRNA screens as a host factor required for HIV infection, specifically the nuclear import of preintegration complexes (PICs) [88,89]. More recently, HIV-1 CA was shown to directly bind the CLD of RanBP2 and this interaction may influence PIC import as well as the integration preference of the viral genome [90,91]. Unlike the CyPA-CA interaction, the RanBP2-CA interaction is insensitive to CsA. Surprisingly, CyPA depletion in RanBP2 knockdown cells rescued HIV infectivity, supporting a role of these two CA-binding cyclophilins in the same nuclear import pathway [90]. These results are consistent with previous studies which implicated CyPA in nuclear transport [92,93,94]. Indeed, as suggested by Schaller *et al.* [90], CLD-containing proteins may act to regulate timing of critical events. In this case, the authors propose monomeric CyPA may act to stabilize the HIV-1 CA until it reaches the nuclear pore whereas the multiple copies of RanBP2 present in the NPC promote uncoating.

## 4. Cyclophilins and HCV

Following the reports of a direct anti-HCV effect of CsA in the HCV replicon systems [95,96], an essential role of cyclophilins in HCV replication was further supported by the correlation between the ability of the different CsA derivatives to bind cyclophilins and to inhibit HCV [97]. The identity of the specific isoform of cyclophilin most important for HCV replication, however, was initially controversial [97,98,99] until several independent groups demonstrated that CyPA is universally and specifically required for HCV infection [100,101,102,103,104]. This was perhaps not surprising when considering the high expression of the CyPA isoform relative to other members of the CyP family. It was also demonstrated that the PPIase motif of CyPA is required for it to function as a HCV cofactor [101,102,103]. Intriguingly, SNPs in the PPIA gene that destabilize CyPA can protect hepatocytes from HCV infection in cell culture [105], suggesting PPIA SNPs contribute to the observed heterogeneity of HCV permissiveness in hepatoma cell populations *in vitro*.

The precise roles of CyPA in the HCV life cycle, and therefore the antiviral mechanisms of CsA and its derivatives, are still not fully understood. CyPA has been proposed to stabilize the HCV replication complex through binding of the HCV replicase [103]; to modulate the RNA-binding properties of the nonstructural protein 5A (NS5A) [106,107]; to act as a cofactor for the RNA‑dependent RNA polymerase [97]; and to influence processing of HCV polyproteins [101]. CyPA may also play a role in more than one step of the HCV infection cycle. Early CsA inhibition studies [95,96,100] used subgenomic replicon assays which only measure RNA replication but not other steps such as entry or assembly. The subsequent observation of differences in the cyclophilin inhibitor (CPI) sensitivity between subgenomic replicons and full-length viruses suggested a function of cyclophilins in an additional step such as viral assembly [104]. Determining a specific role of CyPA in HCV assembly had been difficult, however, due to the fact that replication is a prerequisite for assembly in the natural infection cycle. To overcome this obstacle, Nag *et al.* devised an approach to normalize RNA replication first, and then, specifically examine assembly in CyPA knockdown cells [107]. These results demonstrated that CyPA indeed plays an additional role at the assembly step of HCV life cycle. A recent report showed that CyPA could also interact with the interferon (IFN) regulatory factor 9 (IRF9) [108] *in vitro*. However, because previous studies have demonstrated that CsA functions through an IFN-independent mechanism [95,99], the significance of the CyPA-IRF9 interaction remains unclear. 

An involvement of NS5A in CyPA’s action was first implicated by an analysis of mutations that conferred CsA resistance to HCV replicon *in vitro* [109] and later confirmed by the demonstration of a direct interaction between recombinant NS5A domain II and CyPA [110]. Even though other viral proteins, including NS5B and NS2, have also been suggested to be targets of CyPA, the NS5A-CyPA interaction has the strongest experimental support [110,111,112,113], and, in contrast to the well-defined HIV‑1 CA CyPA-binding site, multiple proline residues serve as the putative substrates for CyPA’s PPIase activity in domains II and III of NS5A [110,111,114,115]. Elucidating the structural changes of NS5A induced by CyPA-binding and isomerization is the next logical step but will be challenging because of the intrinsically disordered nature of these domains [116,117]. 

Despite the strong inhibitory effect of CsA *in vitro*, the *in vivo* effect of CsA on HCV infection and disease progression is less clear, at least in liver transplant patients. Direct comparison of patients who received either CsA or Tacrolimus (FK506), the latter of which did not have any anti-HCV effect *in vitro*, failed to show significant difference in the severity of recurrent HCV in most studies [118,119,120,121]. On the other hand, combination of IFN-a and CsA was more effective in achieving sustained virologic response than IFN-a monotherapy, especially in patients with genotype 1 virus, high viral load, or both [122,123]. More interestingly, clinical trials of CsA derivatives lacking immunosuppressive function have shown that these compounds can potently suppress HCV viral load in patients and may become part of the future combination therapy against HCV [124,125]. Several reasons could account for the differences between the transplant patient results and the positive clinical studies. First, the immune functions of the transplant patients are suppressed by CsA and the antiviral effect alone may not be enough to suppress the virus; all the anti‑HCV clinical CPIs lack the immunosuppressive function. Second, the standard dosage of CsA used in the transplant patients was too low as far as the anti-HCV function is concerned. The leading CPI, (Alisporivir), which was already more potent than CsA at inhibiting HCV replication *in vitro*, was given at least twice the daily dosage, and more frequently, than that of CsA in liver transplant patients. Finally, sequence polymorphisms flanking conserved regions of NS5A may affect *in vivo* susceptibility to CsA [150]. Development of non-CsA-derived CPIs may offer structural diversity in this class of host-targeting compounds [126]. 

In vitro HCV resistance to CPIs has been reported variously and recent data point to a conserved, proline-rich region in NS5A domain II as the hotspot for developing resistance mutations [111,113,127,128]. These mutations do not affect the affinity of NS5A for CyPA and may instead function by promoting a conformational tendency naturally induced by CyPA binding [111]. Unlike viral resistance to direct antiviral agents (DAAs), the magnitude of the *in vitro* resistance to CPIs is relatively low (less than 20 fold as opposed to thousands of folds). Consistent with this, the genetic barriers that CPIs poses in clinical studies are also higher than DAAs [129]. A common concern for targeting a host protein is the associated cellular toxicity; however, in the case of CyPA, knockout studies both in a human cell line and mice suggest CyPA is dispensable for basic cell survival [58,130].

## 5. Cyclophilins and Other Viruses

The potential role of cyclophilins in the replication of influenza virus has been detailed in a separate review in this special issue [131]. In yeast, the CyPA homolog Cpr1p inhibits viral replication proteins of tombusvirus [132]. Interestingly, a parvulin PPIase could also inhibit tombusvirus in this model, supporting overlapping functionality among PPIase classes. West Nile virus RNA and its nonstructural protein 5 (NS5) were found to interact directly with CyPA in infected cells in a CsA‑sensitive manner [133], although the CsA was much less active in inhibiting WNV than HCV. The replication and pathogenesis of many other human viruses have been reported to be regulated either by CsA or cyclophilins. These include polyomavirus BK [134], arterivirus [135], rotavirus [136], human cytomegalovirus [137], vesicular stomatitis virus [138], vaccinia virus [139,140], measles virus [141], human papillomavirus [142,143], coronaviruses [144,145,146], and hepatitis B virus [147,148,149].

## 6. Concluding Remarks

While the diversity of potential functions of host cyclophilins in viral life cycles is evident, what is less apparent is the conservation of these roles. For example, whether most cyclophilin-virus interactions are unique to particular viruses or part of larger, broad themes is uncertain. Furthermore, the cellular and viral substrates of cyclophilins are not fully defined, nor are the implications of catalytically altering the conformation of folded protein. CLD-containing proteins are found in all domains of life and are even found within the genome of at least one virus, the mimivirus [7,8], highlighting the conserved and critical role cyclophilins likely play in the host cell. Further research effort towards understanding the role cyclophilins play in both viral life cycles and normal cell physiology will expand our understanding of the cell as a whole and reveal new host drug targets.
